# Retinal Vessel Diameter and Chronic Kidney Disease in Rural China: A Cross-Sectional Study

**DOI:** 10.1097/MD.0000000000002076

**Published:** 2015-12-11

**Authors:** Shumin Bao, Wen Huang, Yuanbo Liang, Liping Jiang, Fenghua Wang, Yi Peng, Guangjuan Zhang, Ningli Wang

**Affiliations:** From the Department of Nephrology (SB, WH, LJ, GZ), and Beijing Institute of Ophthalmology, Beijing Tongren Eye Center, Beijing Tongren Hospital, Capital Medical University, and Beijing Key Laboratory of Ophthalmology and Visual Sciences, Beijing, China (YL, FW, YP, NW).

## Abstract

Few studies have examined the relationship between retinal microvascular abnormalities and chronic kidney disease (CKD). This study aims to examine the association between retinal vessel diameters and CKD in the rural China in order to provide the scientific basis for the early detection and diagnosis for CKD.

Participants and data were extracted from the Handan Eye Study, a population-based cross-sectional study performed from 2006 to 2007. The central retinal arteriolar equivalent (CRAE) and central retinal venular equivalent (CRVE) were summarized by the average arteriolar and venular caliber of each eye. The estimated glomerular filtration rate (eGFR) and a urinary albumin to creatinine ratio (ACR) were recorded. Multivariate logistic regression models were used to determine any associations between CRAE, CRVE, arteriole-to-venule ratio (AVR), retinopathy, and CKD in the recruited participants.

CKD was found in was 17.3% (892/5158) of this population with a 0.9% (48/5545) rate of reduced renal function and 16.7% (922/5538) rate of albuminuria. Retinopathy was present in 9.6% (571/5925) of participants. Compared to the 4th quartile of AVR, the first group was found to have a higher risk of albuminuria (odds ratio [OR] = 1.261, 95% confidence interval [95%CI]: 1.015–1.567, *P* = 0.037) and CKD (OR = 1.240, 95%CI: 1.000–1.537, *P* = 0.049) after adjustment for potential confounding variables. Retinopathy was associated with the occurrence of albuminuria (OR = 1.340, 95%CI: 1.067–1.685, *P* = 0.012) and CKD (OR = 1.341, 95%CI: 1.071–1.681, *P* = 0.010). In participants with diabetes, the ORs for the 1st and 4th quartiles of CRAE and CRVE were 2.292 (95%CI: 1.076–4.881, *P* = 0.032) and 2.113 (95%CI: 1.006–4.438, *P* = 0.048), respectively. Among the participants with hypertension, retinopathy was also observed to be associated with CKD (OR = 1.306, 95%CI: 1.003–1.699, *P* = 0.047).

The parameters of retinal vessel diameter may be a useful index evaluating the occurrence and development of CKD.

## INTRODUCTION

Chronic kidney disease (CKD) has become a major public health problem worldwide and has been associated with premature morbidity and mortality.^1–3^ CKD has long been a clinically challenging due to the late appearance of its clinical symptoms and the initiation of standard therapies only in late stages of the disease. Therefore, early detection, diagnosis, and treatment are the cornerstones of CKD management.

Some epidemiological studies have shown a relationship between CKD retinal microvascular abnormalities. Risk factors for microvascular damage, such as diabetes and hypertension, have been strongly associated with CKD,^4–7^ and microvascular alterations in renal circulation in CKD have been documented in animal models.^8^ Thus, microvascular disease has been suggested to play a major role in the etiology and evolution of CKD.

However, epidemiological studies are generally focused on urban and economically developed areas, while little is known about rural areas where lifestyle, level of education, cultural customs, nutrition, and environmental factors are very different.^9–11^ Since rural population accounts for the vast majority of China's total populace, exploring the relationship between retinal microvascular abnormalities and CKD in this population is of great significance.

This study examines the association between retinal vessel diameters and CKD in rural China in order to provide a scientific basis for the early detection and diagnosis for CKD.

## MATERIALS AND METHODS

### Study Population

All patient data were extracted from the Handan Eye Study, a population-based cross-sectional study performed from 2006 to 2007. A detailed explanation of this study and its methodology was published by Liang et al in 2009.^9^ Residents of Yongnian County in Handan City, Hebei Province aged 6–30 years were randomly selected using a stratified, clustered, and multistaged sampling technique. Of the 458 villages in this region, 13 were randomly selected and stratified by geographic landform (plains or hillside). The protocol for this study was reviewed and approved by the Ethics Committee of the Beijing Tongren Hospital in accordance with the guidelines of the Helsinki Declaration, and written informed consent was obtained from all participants. Participants with missing information on serum creatinine, urinary albumin, or unclear retinal photography were excluded.

### Data Collection

The information collected from participants included sociodemographic status (age, sex, and education), past medical history (especially history of hypertension and/or diabetes), and lifestyle information (including assessment of smoking and drinking). Physical examinations were performed to measure height, weight, and blood pressure. Blood samples were obtained and fasting blood glucose, lipid levels, urea nitrogen, urea albumin, and creatinine were measured.^9^ Albuminuria was measured by immunoturbidimetric methods (Audit Diagnostic, Ireland), and urinary creatinine was measured by the Jaffé kinetic method.

### Retinal Vessel Diameter Measurement

The procedures for retinal photography and the grading of retinal microvascular signs have been described in a precursor study.^9^ Retinal vessel diameters from digital retinal images were measured using a computer-based program by trained graders who were blinded to the status of each participant. The central retinal arteriolar equivalent (CRAE) and central retinal venular equivalent (CRVE) were summarized using the average arteriolar and venular caliber of each eye. This data were obtained by measuring all arterioles and venules coursing through a zone between 0.5 and 1 disc diameters away from the margin of the optic disc.^10^

Retinopathy was considered present if any characteristic lesion, as deﬁned by the ETDRS severity scale, was present including microaneurysms, hemorrhages, cotton wool spots, intraretinal microvascular abnormalities, hard exudates, venous beading, and/or new vessels.

### Definition of CKD

CKD was deﬁned as the presence of either reduced renal function as evaluated by estimated glomerular filtration rate (eGFR, <60 mL/minute/1.73 m^2^) or albuminuria evaluated by urinary albumin to creatinine ratio (ACR, ≥30 mg/g).^11^ The eGFR was calculated using a modified version of the Modification of Diet in Renal Disease equation developed by the Peking University First Hospital, based on data from Chinese patients with CKD.^12^

### Definition of Other Variables

Hypertension was defined as systolic blood pressure ≥140 mmHg and diastolic blood pressure ≥90 mmHg, use of antihypertensive medication, or a self-reported previously diagnosed hypertension. Diabetes mellitus was defined as fasting blood glucose ≥7.0 mmol/L, a self-reported history of diabetes, or those currently receiving insulin or oral hypoglycemic agents.

### Statistical Analysis

Body mass index (BMI) was calculated (kg/m^2^) and participants were classified into either an overweight/obesity group (≥24.0 kg/m^2^) or a normal/underweight group (<24.0 kg/m^2^). Level of education was based on number of years of schooling and classified into 2 groups, ≥9 and <9 years. The arteriole-to-venule ratio (AVR) was calculated as the ratio of CRAE to CRVE. The parameters of CRAE, CRVE, and AVR were categorized into quartiles.

Population characteristics were described using means and standard deviations (SDs) or frequencies and proportions. The characteristics of the different quartiles of CRAE, CRVE, and AVR were also summarized and tested with one-way analysis of variance or the Chi-square test where appropriate. Multivariate logistic regression models were used to determine if associations existed between CRAE, CRVE, AVR, and/or retinopathy and CKD in the recruited population. Separate analyses were also performed for participants with diabetes, hypertension, and obesity. The effects of potential confounding variables and bias were controlled by adjusting the age, sex, BMI, education, hypertension, diabetes, smoking, drinking, total cholesterol, triglyceride, low-density lipoprotein (LDL), and high-density lipoprotein (HDL) levels.

Odds ratios (ORs) and their 95% confidence intervals (95%CIs) were estimated using the maximum likelihood method. A 2-sided *P* value of <0.05 was considered to be statistically significant. All analyses were performed using SAS software, version 9.1.3 (SAS Institute, Cary, NC).

## RESULTS

A total of 5925 participants were recruited into the study. Retinal imaging and retinal vessel diameter measurements were obtained for all participants. eGFR data were available for 5158 participants and albuminuria data for 5538.

In this population, CKD had a prevalence of 17.3% (892/5158), reduced renal function in 0.9% (48/5545), albuminuria in 16.7% (922/5538), and retinopathy in 9.6% (571/5925) of participants. Basic characteristics of the participants are shown in Table 1. Participants with CKD were mostly older females and had greater incidences of hypertension, diabetes, and smoking (*P* < 0.05). Total cholesterol, triglycerides, and LDL levels were also found to be higher than in the general population (*P* < 0.05).

**TABLE 1:**
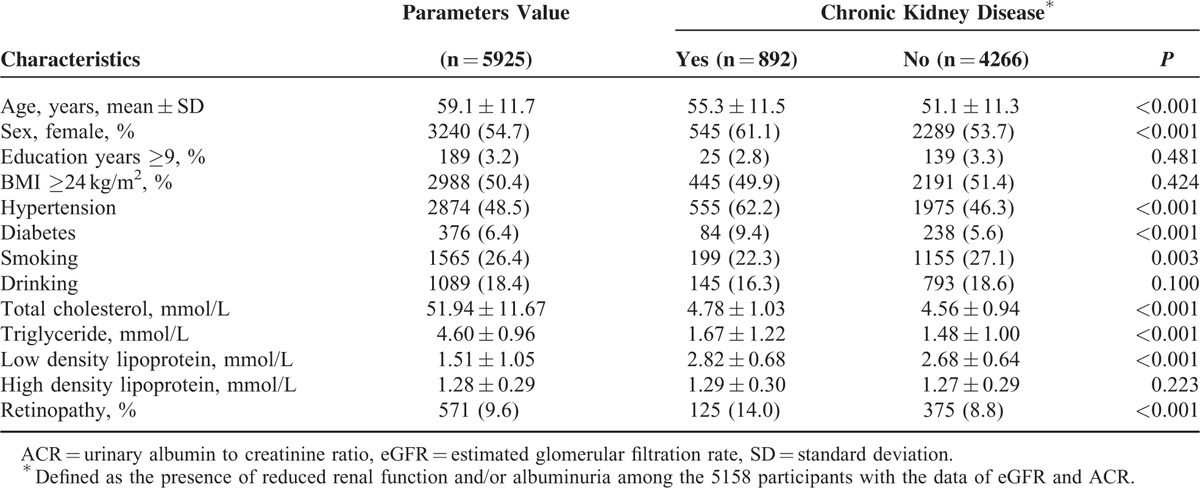
The Basic Characteristics of the Participants Recruited in the Study

The differences between the CRAE quartiles were statistically significant for age, BMI, hypertension, diabetes, smoking, total cholesterol, LDL levels, and HDL levels (*P* < 0.05, Table 2). Sex, BMI, diabetes, smoking, alcohol consumption total cholesterol, triglycerides, LDL levels, and HDL levels revealed significant differences in the CRVE quartiles (*P* *<* 0.05, Table 3). The proportions of obesity, hypertension, diabetes, and mean total cholesterol, triglyceride, and LDL levels decreased with the increased AVR quartiles (*P* *<* 0.05, Table 4).

**TABLE 2:**
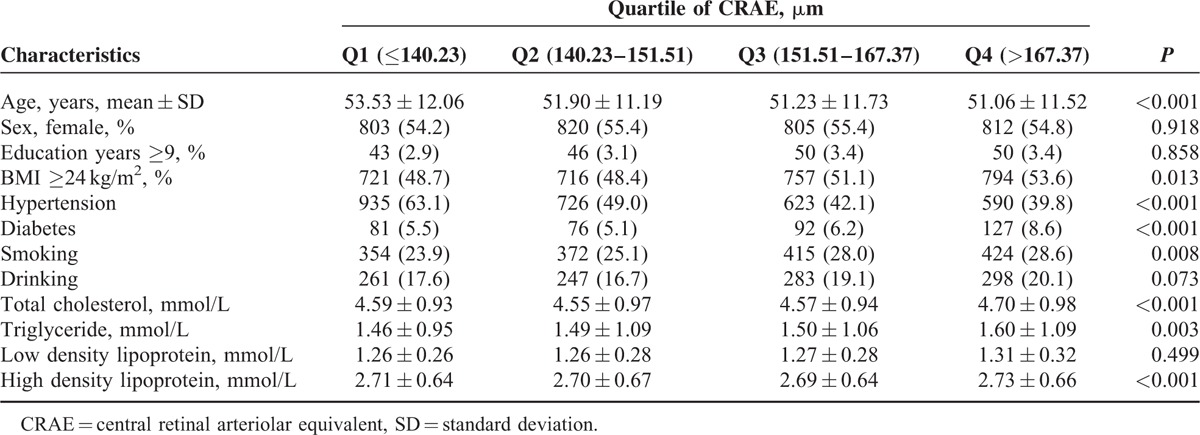
The Population Characteristics Grouped by Quartiles of Central Retinal Arteriolar Equivalent

**TABLE 3:**
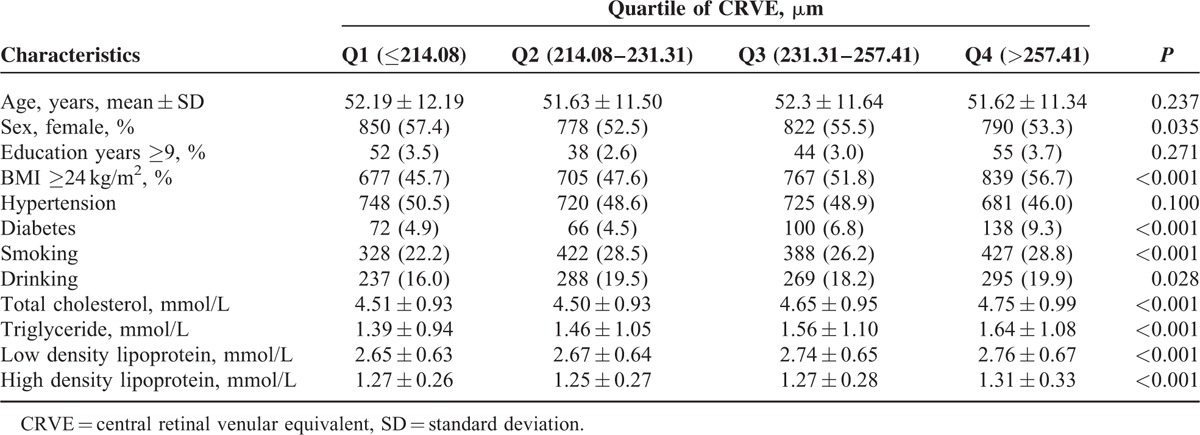
The Population Characteristics Grouped by Quartiles of Central Retinal Venular Equivalent

**TABLE 4:**
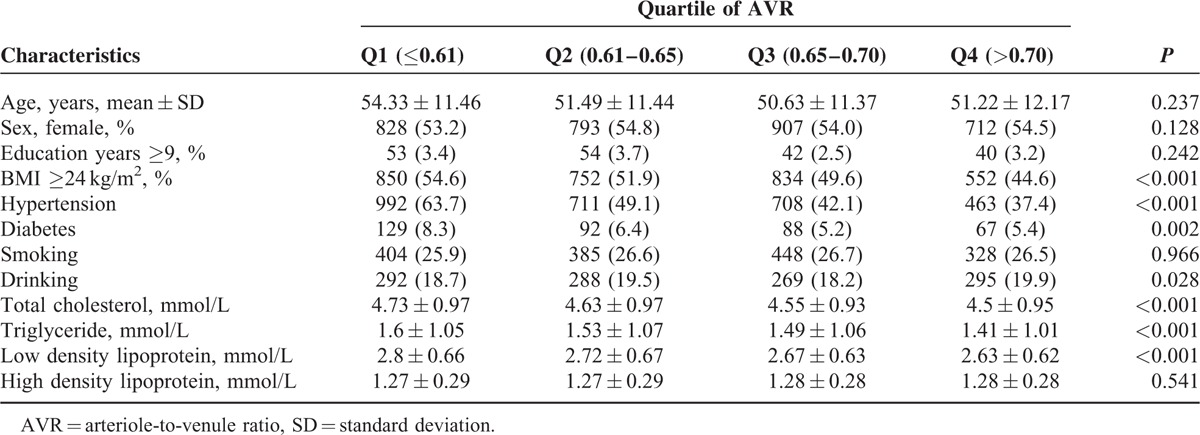
The Population Characteristics Grouped by Quartiles of Arteriole-to-Venule Ratio

The associations between CRAE, CRVE, AVR, and CKD were analyzed with multivariate logistic regression models (Table 5). Compared to the 4th AVR quartile, the first quartile had a higher risk of albuminuria (OR = 1.261, 95%CI: 1.015–1.567, *P* = 0.037) and CKD (OR = 1.240, 95%CI: 1.000–1.537, *P* = 0.049) when adjusted by age, sex, incidence of smoking, alcohol consumption, BMI, level of education, and total cholesterol, triglycerides, and LDL, and HDL levels. Retinopathy was also associated with the occurrence of albuminuria (OR = 1.340, 95%CI: 1.067–1.685, *P* = 0.012) and CKD (OR = 1.341, 95%CI: 1.071–1.681, *P* = 0.010).

**TABLE 5:**
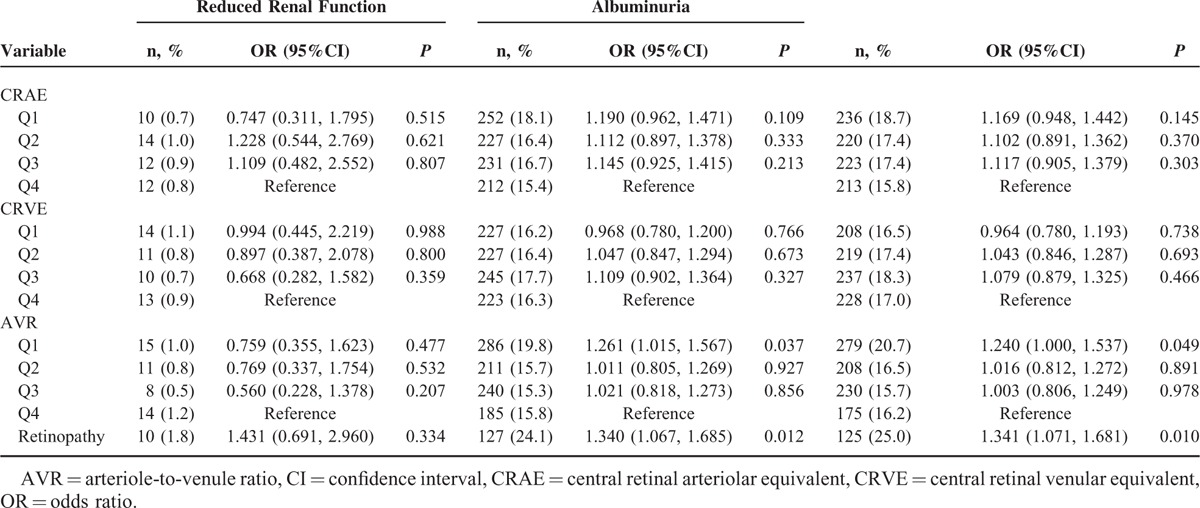
The Associations Among Central Retinal Arteriolar Equivalent, Central Retinal Venular Equivalent, Arteriole-to-Venule Ratio and Chronic Kidney Disease in All The Participants

In participants with diabetes, the ORs for CKD in the first CRAE and CRVE quartile 2.292 (95%CI: 1.076–4.881, *P* = 0.032) compared with 2.113 (95%CI: 1.006–4.438, *P* = 0.048) in the 4th quartile (Table 6). Among participants with hypertension, retinopathy was also found to be associated with CKD (OR = 1.306. 95%CI: 1.003–1.699, *P* = 0.047), as shown in Table 7. No significant differences were found between CRAE, CRVE, ACR, and CKD in participants with obesity (Table 8).

**TABLE 6:**
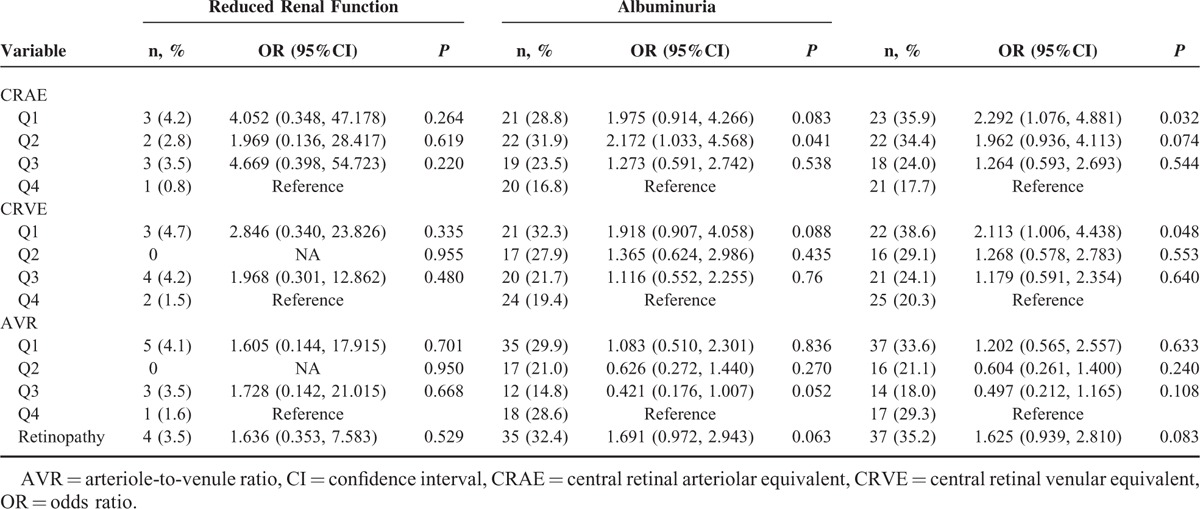
The Associations Among Central Retinal Arteriolar Equivalent, Central Retinal Venular Equivalent, Arteriole-to-Venule Ratio and Chronic Kidney Disease in the Participants With Diabetes

**TABLE 7:**
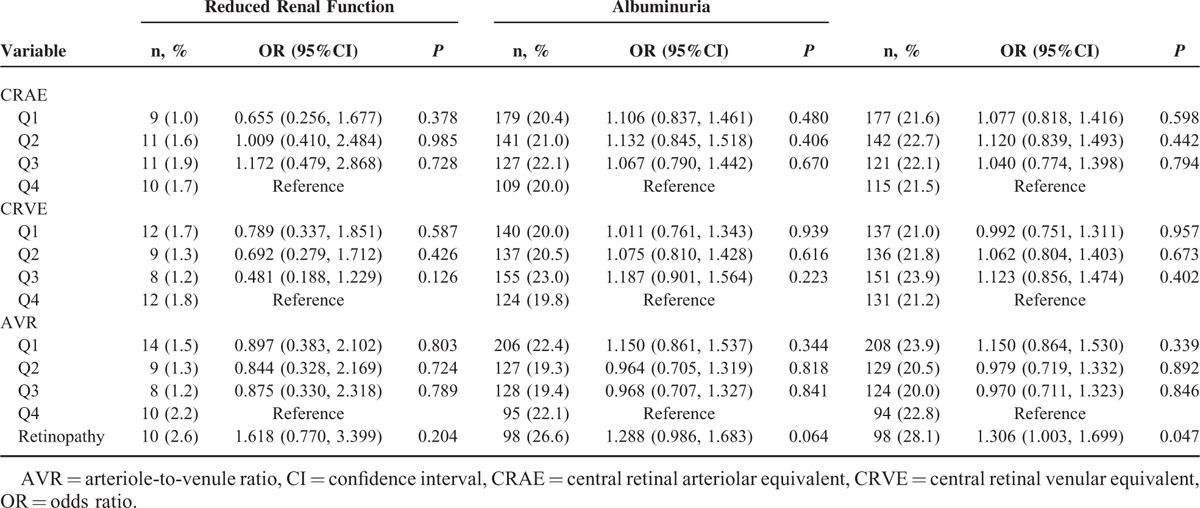
The Associations Among Central Retinal Arteriolar Equivalent, Central Retinal Venular Equivalent, Arteriole-to-Venule Ratio and Chronic Kidney Disease in the Participants With Hypertension

**TABLE 8:**
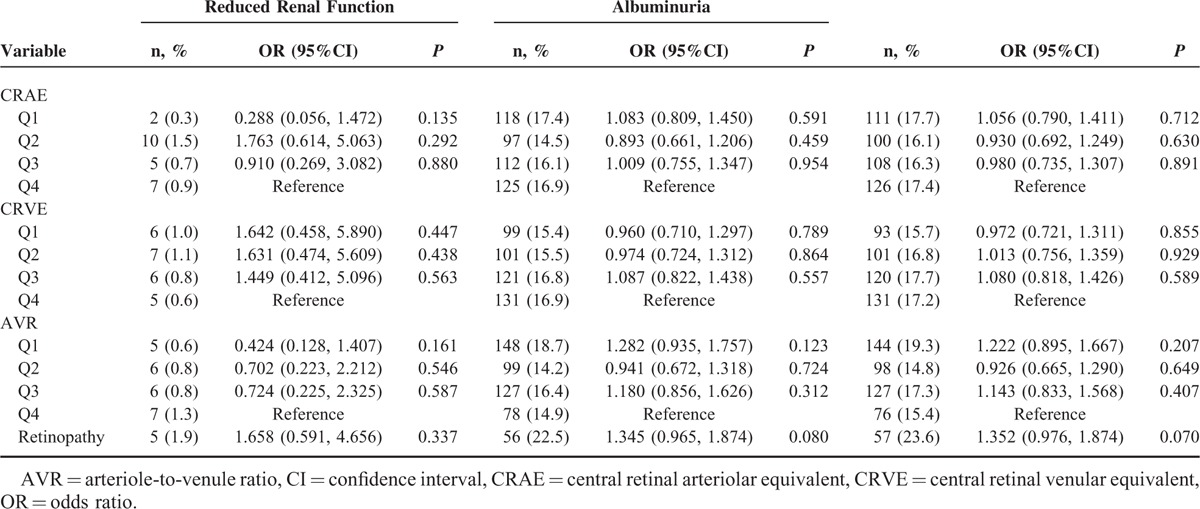
The Associations Among Central Retinal Arteriolar Equivalent, Central Retinal Venular Equivalent, Arteriole-to-Venule Ratio and Chronic Kidney Disease in the Participants With Overweight/Obesity

## DISCUSSION

Retinal microvasculature provides a unique window for noninvasive visualization of human circulation in vivo. Retinal microvasculature is considered as one of the best indicators of systemic microcirculation status in humans.^3,6,7,13,14^ In recent years, thanks to the continuous development of fundus photography and digital image analysis technology, several large-scale epidemiological surveys have been conducted in other countries. These studies analyzed the relationship between retinal vascular abnormalities and changes in blood pressure, cardiovascular disease, kidney disease, and/or other systemic diseases.^3,4,7,14–17^ Few studies reported a relationship between retinal vascular abnormalities and CKD, although the results were entirely comparable.^6^

According to the Atherosclerosis Risk in Communities Study, people with retinopathy were more likely to suffer from CKD (OR = 2.0; 95%CI: 1.4–2.8) after adjustment for potential confounding factors.^6^ In the Singapore Malay Eye Study (SiMES), retinopathy was related to a decrease in eGFR and albuminuria.^7^ In our analysis, we found a weaker association between retinopathy and CKD compared to the above studies.

In the Chronic Renal Insufficiency Cohort study, presence and severity of retinopathy at baseline were strongly associated with the risk of subsequent progression to end-stage renal disease and reductions in eGFR in univariate analyses. However, this association was not statistically significant after adjustment for initial eGFR and 24-hour proteinuria.^18^ Adjustment for all potential confounding factors is extremely important in order to determine the association between retinopathy and CKD, and further studies may be warranted.

In this study, we did not find an association between quartiles of CRAE and CRVE and CKD considering the whole sample or just hypertensive participants. The presence of retinal arteriolar abnormalities was also not associated with deteriorating renal function in the Cardiovascular Health Study.^19^ On the other hand, in the Atherosclerosis Risk in Communities Study, patients with retinal arteriolar/venular narrowing were more likely to suffer from CKD compared to the general population. In the SiMES, only retinal arteriolar narrowing was related to increased albuminuria, while no difference was found for retinal venular changes.^7^

Interestingly, we found that the likelihood of suffering from CKD increased with decrease in retinal diameter. Therefore, AVR may act as a marker for the early stages of CKD. However, this association could not be observed once the patients were stratified based on hypertension and diabetes diagnoses. This confirms the results obtained by Masaidi et al^20^ who reported that no significant associations could be found between AVR and eGFR/microalbuminuria in hypertensive patients. However, our results could be explained by the specificity of this population.

The association between CKD/albuminuria and retinopathy has been evaluated innumerousstudies.^13,16,21–23^ For example, the SiMES showed that retinal arteriolar narrowing was associated with CKD.^19^ We did not find statistically significant associations between these variables in the general population, but we did find that retinal arterial or venular narrowing increases as CKD progresses in patients with diabetes. Previous studies have also reported that type 2 diabetic patients with proliferative diabetic retinopathy are more likely to present with renal involvement.^23^ Therefore, all patients with proliferative diabetic retinopathy or with retinal arterial or venular narrowing should undergo an evaluation of renal function, including urinary albumin measurements.

Zhang et al^24^ reported that the prevalence of CKD was 14.3% in 13 provinces in China – not including Hebei province analyzed herein – with 2.8% of the population having reduced renal function and 7.3% having albuminuria. Xue et al^25^ sampled residents of Guangxi Province and reported that the prevalence of albuminuria and reduced renal function were 12.5% and 0.4%, respectively – the overall CKD prevalence was 15.3%. Compared to these studies, we have found similar prevalence of CKD but a higher rate of albuminuria which may be related to lifestyle differences.

The main limitation of this study is its cross-sectional nature which limits determination of causal inferences. Secondly, albuminuria was measured only once using urinary ACR, which may overestimate or underestimate the real prevalence of albuminuria. Additionally, a selection bias may have occurred due to exclusion of eligible participants with incomplete records. The results from this population-based data from rural China suggest that retinal vessel diameter is associated with CKD, especially in the diabetic population, and may serve as a useful index for evaluating the occurrence and development of CKD.
